# The Precise Breakpoint Mapping in Paracentric Inversion 10q22.2q23.3 by Comprehensive Cytogenomic Analysis, Multicolor Banding, and Single-Copy Chromosome Sequencing

**DOI:** 10.3390/biomedicines10123255

**Published:** 2022-12-14

**Authors:** Tatyana V. Karamysheva, Tatyana A. Gayner, Eugeny A. Elisaphenko, Vladimir A. Trifonov, Elvira G. Zakirova, Konstantin E. Orishchenko, Mariya A. Prokhorovich, Maria E. Lopatkina, Nikolay A. Skryabin, Igor N. Lebedev, Nikolay B. Rubtsov

**Affiliations:** 1 Institute of Cytology and Genetics, Siberian Branch of Russian Academy of Sciences (SB RAS), 630090 Novosibirsk, Russia; 2 Institute of Chemical Biology and Fundamental Medicine, Siberian Branch of Russian Academy of Sciences (SB RAS), 630090 Novosibirsk, Russia; 3 Group of Companies, Center of New Medical Technologies, 630090 Novosibirsk, Russia; 4 Institute of Molecular and Cellular Biology, Siberian Branch of Russian Academy of Sciences (SB RAS), 630090 Novosibirsk, Russia; 5 Department of Genetic Technologies, Novosibirsk State University, 630090 Novosibirsk, Russia; 6 Tomsk National Research Medical Center of the Russian Academy of Sciences, Research Institute of Medical Genetics, 634050 Tomsk, Russia

**Keywords:** paracentric inversion, inv(10), breakpoint mapping, array-based comparative genomic hybridization, chromosomal microdissection, multicolor banding, single-copy chromosome sequencing, reproductive failure, oligoasthenoteratozoospermia

## Abstract

Detection and precise genomic mapping of balanced chromosomal abnormalities in patients with impaired fertility or a clinical phenotype represent a challenge for current cytogenomics owing to difficulties with precise breakpoint localization in the regions enriched for DNA repeats and high genomic variation in such regions. Here, we present a comprehensive cytogenomic approach to breakpoint mapping in a rare paracentric inversion on 10q (in a patient with oligoasthenoteratozoospermia and necrozoospermia) that does not affect other phenotype traits. Multicolor banding, chromosomal microarray analysis, chromosome microdissection with reverse painting, and single-copy sequencing of the rearranged chromosome were performed to determine the length and position of the inverted region as well as to rule out a genetic imbalance at the breakpoints. As a result, a paracentric 19.251 Mbp inversion at 10q22.2q23.3 was described. The most probable location of the breakpoints was predicted using the hg38 assembly. The problems of genetic counseling associated with enrichment for repeats and high DNA variability of usual breakpoint regions were discussed. Possible approaches for cytogenomic assessment of couples with balanced chromosome rearrangements and problems like reproductive failures were considered and suggested as useful part of effective genetic counseling.

## 1. Introduction

Balanced chromosome rearrangements may have no pathogenic impact or be associated with various phenotypic abnormalities. The reasons for abnormalities could be gene damage or a disturbance of the genome structure responsible for the regulation of gene expression. Such a disturbance may result from a small deletion, insertion, other DNA changes in breakpoint regions, or a structural disturbance of topologically associating domains (TADs). The identification of the changes in breakpoint regions is often difficult. We believe that the estimation of the power of various techniques applied to cytogenomic analysis is important for the correct diagnosis of balanced chromosome rearrangements. Inversions are among the most convenient chromosomal abnormalities for such estimation. They constitute ~10% of the structural chromosomal aberrations in humans [1]. Inversions may form without a genome imbalance or may be accompanied by an imbalance that is undetectable by routine diagnostics.

For an inversion, two breaks on the same chromosome followed by an insertion of the cut-out chromosome fragment in the opposite orientation are required [1]. There are two types of inversions: pericentric and paracentric. Pericentric inversions involve short and long chromosome arms (p-arm and q-arm, respectively), and the inverted regions include the centromere. Paracentric inversions occur in one of the chromosome arms [2]. According to recent studies, paracentric inversions are some of the most frequent types of chromosome rearrangements. The most common paracentric inversions are inv(3) (prevalence 18.46%), inv(7) (16.92%), inv(11) (10.77%), inv(1) (9.23%), and inv(5) (7.69%) [1]. Their prevalence is probably underestimated. Small paracentric inversions are difficult to identify with routine diagnostics and can remain undetected. Furthermore, very small (submicroscopic) paracentric inversions usually can be distinguished from normal human genomic variation. Regarding the clinical effects of large and small/submicroscopic paracentric inversions, see ref. [3] and the database of genomic variants (http://dgv.tcag.ca/dgv/app/home accessed on 5 November 2022).

Besides the possible problems associated with changes in the regions where the breaks and fusion took place, there is a risk of unequal events of crossing over inside the inverted chromosome region. Their risk can be estimated with the genetic distance between the breakpoint regions considering the phenomenon of crossover suppression by inversion. The greater genetic distance between these regions means a higher risk of a crossover inside the inverted chromosome regions, resulting in a higher risk of gametes with a genomic imbalance [4,5]. A crossover does not take place in small inverted regions. Usually, the high probability of a crossover is present if an inverted region represents at least 30% of chromosome arm length [4].

Parents with a large enough inversion can produce unbalanced gametes, and there is a risk of offspring with a genomic imbalance [6]. In case of a paracentric inversion and unequal crossover events in the inverted chromosome region, gametes contain either an acentric fragment or a dicentric chromosome. As a result, the mortality rate of embryos that developed with such a genomic imbalance is near 100%. Fetuses having an acentric chromosome fragment or a dicentric chromosome are usually not viable [1]. As for a pericentric inversion, the unequal crossover events in the inverted region can lead to gametes with a deletion of one chromosome region and a duplication of another one. Thus, pericentric inversions can result in live-born children with birth defects in inversion carriers owing to the presence of partial trisomy or partial monosomy. The overall risk of offspring with a genomic imbalance for carriers of a pericentric inversion depends on crossover frequency in the inverted chromosome region and prenatal mortality of embryos having abnormal chromosomes. On average, for a parent carrying a pericentric inversion, the frequency of such children born alive with a genomic imbalance appears to be approximately 5–10% [7]. The genomic imbalance in such a fetus can usually be detected with prenatal diagnostics via standard cytogenetic techniques such as GTG-banding and especially array-based comparative genomic hybridization (aCGH).

Chromosomal abnormalities involving a large chromosome region and resulting in a genomic imbalance usually have phenotypic manifestations. Inversions combined with a detected deletion, duplication, or another type of rearrangement are typically regarded as complex chromosomal abnormalities. Traditionally, rearrangements referred to as inversions have shown no visible imbalance of chromosome regions. Nonetheless, we should mention that finding an answer to the question about small rearrangements in breakpoint regions is difficult. What is the main problem with describing such breakpoint regions in detail? Does it derive from the insufficient resolution of molecular cytogenomic techniques used for the diagnosis of the chromosomal abnormality or from diversity of individual genomes? An assessment of capabilities of the modern molecular cytogenomic techniques widely employed for chromosome diagnostics is important for drawing the right conclusion from the results of a cytogenomic analysis.

Here we studied breakpoint regions of an inversion in the long arm of chromosome 10 in a patient with male infertility. There are numerous possible reasons for male infertility. A genetic abnormality in cases of male infertility has been revealed in 10–15% of couples who have tried to conceive; approximately half of the abnormalities are due to a “male factor” [8]. Nonetheless, in many cases, severe male infertility has a genetic cause including chromosomal aberrations (according to various studies: 4.3% to 9.6% of cases) [9,10,11]. In our work, GTG-banding; different fluorescence in situ hybridization (FISH) techniques, including chromosome microdissection with reverse painting; multicolor banding; aCGH; and single-copy inverted chromosome sequencing were applied for comprehensive breakpoints mapping.

## 2. Materials and Methods

### 2.1. The Clinical Case

The patient—33 years of age, male—got a diagnosis of infertility due to oligoasthenoteratozoospermia and necrozoospermia. Genetic counseling revealed no cognitive or physical abnormalities. No cases of infertility, congenital abnormalities, or intellectual disability were found in his pedigree. The sperm concentration was 8.4 million/mL (the WHO reference is 16 million/mL [12]), and the total sperm count in an ejaculation was 22.68 million. Sperm morphology was assessed after the centrifugation of the entire volume of the ejaculate. Normal morphology was registered for 0% of the sperm, and mild pathologies for 100% of the sperm. Head, midpiece, and tail pathologies were detected in 99%, 39%, and 30% of the sperm, respectively. The teratozoospermia index (TZI) was 1.68. Progressively motile (PR), non-progressively motile (NP) and immotile (IM) spermatozoa accounted for 0% (the reference PR is 30% [12]), 9% and 91%, respectively. The index of total motility (PR+NP) was 9% (the reference PR+NP is 40% [12]). An increase in follicle-stimulating-hormone and luteinizing-hormone levels as well as signs of atopy (i.e., allergy to fur and more rarely to aspirin) were noted.

The patient’s wife had an increased level of 17-hydroxyprogesterone (7.39 μU/mL) and an elevated level of prolactin (18.85 μU/mL). Multifollicular echostructure of the ovaries was detected by ultrasonography. Assisted reproductive technologies were recommended to this couple because of female infertility associated with a male factor.

### 2.2. Chromosome Preparation and GTG-Banding Analysis

The chromosomal analysis of the patient and his wife was performed on phytohemagglutinin-stimulated peripheral blood lymphocytes. The lymphocytes were cultured with a standard protocol for preparation fixed metaphases [13]. Then, the cultured cells were treated with a hypotonic solution (0.075 M KCl; Sigma-Aldrich, Saint Louis, MO, USA) and fixed in a cold methanol:glacial acetic acid mixture (3:1). The cytogenetic evaluation of metaphase chromosomes was performed with the GTG-banding technique (G-bands by trypsin using Giemsa staining) for chromosome identification and a search for chromosome rearrangements [14].

### 2.3. Creation of Microdissected DNA Probes

DNA probes were synthesized with the DNA amplification of microdissected and collected metaphase chromosomes (whole chromosome paint: WCP) or chromosome regions (partial chromosome paint: PCP) according to a standard protocol [15]. Micromanipulation procedures were performed under an Axiovert 10 inverted microscope (objective 100×, ocular 10×; Zeiss, Aalen, Germany) and a glass needle controlled by an MR micromanipulator (Zeiss, Aalen, Germany). Next, the isolated material was transferred into a 20–60 nL collection drop (10 mM Tris-HCl pH 7.5, 0.1% of SDS, 0.1% of Triton X-100, 30% of glycerol, and 0.5 mg/mL proteinase K) placed into an extended siliconized tip of a Pasteur pipette. The dissected material placed in the collection drop was incubated for 2 h at 60 °C in a water bath. After that, the DNA samples were amplified with degenerate oligonucleotide primed PCR with the MW6 primer (5′-CCG ACT CGA GN_6_ATG TGG-3′) [16,17]. DNA labeling with TAMRA-dUTP (Roche, Basel, Switzerland), Alexa Fluor 488-dUTP (Invitrogen, Waltham, MA, USA), or DEAC–dUTP (Jena Bioscience, Jena, Germany) was performed according to standard protocols [17].

The quality of the prepared WCPs and PCPs was verified with chromosomal in situ suppression (CISS) hybridization on metaphase chromosomes of the patient and a healthy donor [18]. For chromosome identification after CISS hybridization, chromosomes were stained with 4′,6-diamidine-2′-phenylindole dihydrochloride (DAPI) (Sigma-Aldrich, Saint Louis, MO, USA) [13], and inverted DAPI-banding was analyzed. ISCN 2020 was employed for chromosome description [19].

### 2.4. Multicolor Chromosome Banding (MCB)

For describing chromosome regions on the basis of their DNA content, MCB was applied [20,21]. Three PCPs were prepared from microdissected DNA libraries generated from chromosome regions 10q11–q21 (PCP10A), 10q21–q23 (PCP10B), and 10q23–q25 (PCP10C) [22]. The dissection was performed in a way to obtain the maximal copy number of the fragments containing a central part of the dissected region [20,21,22]. PCPs were prepared by labeling with TAMRA-dUTP (PCP10A), Alexa Fluor 488-dUTP (PCP10B), or DEAC-dUTP (PCP10C). CISS hybridization with the PCPs was conducted as described elsewhere [15]. Microscopy was carried out with a microscope AxioPlan 2 Imaging (Zeiss, Aalen, Germany) equipped with filter sets No. 49 (Zeiss, Aalen, Germany), SP101 FITC (CHROMA, Wixon, MI, USA), SP103v1 Cy3tmv1 (CHROMA, Wixon, MI, USA), and a CCD camera (CoolCube 1, MetaSystems GmbH, Altlussheim, Germany). The capture of micrographs and their processing were carried out in the ISIS5 software (MetaSystems GmbH, Altlussheim, Germany). For the generated MCB, an appropriate option in the ISIS5 software was chosen.

### 2.5. aCGH 

This procedure was performed using the SurePrint G3 Human CGH + SNP 4 × 180 K Microarray Kit (4 × 180 K) (Agilent Technologies, Santa Clara, CA, USA) with 13 kbp overall median probe spacing (11 kbp in RefSeq genes) according to the manufacturer’s instructions. Labeling and hybridization of the patient’s DNA and reference DNA (#5190-3796, Human Reference DNA male, Agilent Technologies, Santa Clara, CA, USA) were implemented by enzymatic labeling and hybridization protocols (v.7.5, Agilent Technologies, Santa Clara, CA, USA). Array images were acquired on an Agilent SureScan Microarray Scanner (Agilent Technologies, Santa Clara, CA, USA). Data analysis was performed using the CytoGenomics software (v.3.0.6.6) (Agilent Technologies, Santa Clara, CA, USA) and the publicly available Database of Genomic Variants (DGV) resources [23]. Human genome assembly 19 (hg19) was utilized to describe the molecular karyotype revealed by aCGH.

### 2.6. Sequencing of the Microdissected DNA Library Generated from the Single-Copy of Inv(10)(q?22.3q?23.3) Chromosome 

The microdissected DNA library derived from the chromosome inv(10)(q?22.3q?23.3). On the base of GTG-banding analysis of rearranged chromosome, it was suggested that region 10q22.3→q23.3 was inverted. GTG- banding did not allow the precise identification of the breakpoints but allowed the identification of the rearranged chromosome for its isolation with microdissection technique. We prepared microdissected DNA library from a single copy of the rearranged chromosome by DNA amplification with DOP-PCR and subsequent se-quencing. A KAPA adapter ligation and library preparation kit (KAPA Single-Indexed Adapter Kit Illumina^®^ Platforms KR1317—v3.17, Saint Louis, MO, USA) was applied. The quantity and quality of the library were assessed by means of an Agilent Bioanalyser, followed by next-generation sequencing (NGS) on the Illumina X Ten platform. The resulting 150 bp paired-end reads were aligned with the human genome (hg38) in HISAT2 [24] and visualized in GENEIOUS [25]. Human genome assembly GRCh38 was used to describe the content of chromosome inv(10)(q?22.3q?23.3) revealed by NGS.

### 2.7. Ethics

The study protocol was approved by the Research Ethics Committee of the Research Institute of Medical Genetics, Tomsk National Research Medical Center (decision No. 8 of 30 November 2020). The patient and his wife were enrolled in the study in strict accordance with international standards, including informed consent and confidentiality. All analyses were in compliance with ethical standards based on the Helsinki Declaration of the International Medical Association with the year 2000 amendments. The patient and his wife signed informed consent forms for the use of their respective biological samples for scientific purposes.

## 3. Results

### 3.1. Routine Karyotyping

**Figure 1:**
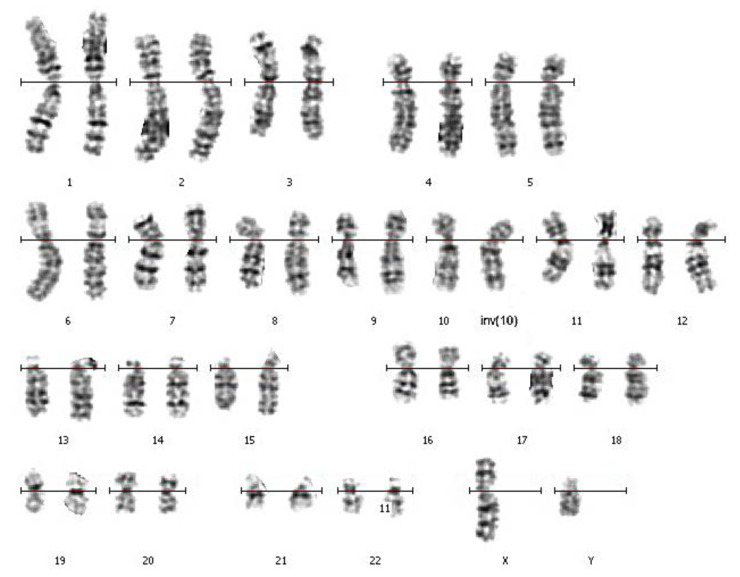
The patient’s GTG-banded chromosomes. The rearranged homolog of chromosome 10 is marked with “inv(10)”.

This analysis of the patient and his wife was carried out by the GTG-banding technique in the Cytogenetic Laboratory at the Center of New Medical Technologies (Novosibirsk, Russia). It revealed an inversion in the q arm of chromosome 10 of the patient’s karyotype (Figure 1). The patient’s karyotype was described as 46,XY,inv(10)(q?22.3q?23.3). The karyotype of his wife was described as 46,XX with the help of GTG-banding.

The parents of the patient and his other relatives were not available for the cytogenetic analysis. The obtained karyotype description required confirmation of the revealed inversion with a more precise mapping of the inversion breakpoints and testing for possible deletions in the breakpoint regions.

### 3.2. CISS Hybridization with PCPs and MCB Analysis 

**Figure 2:**
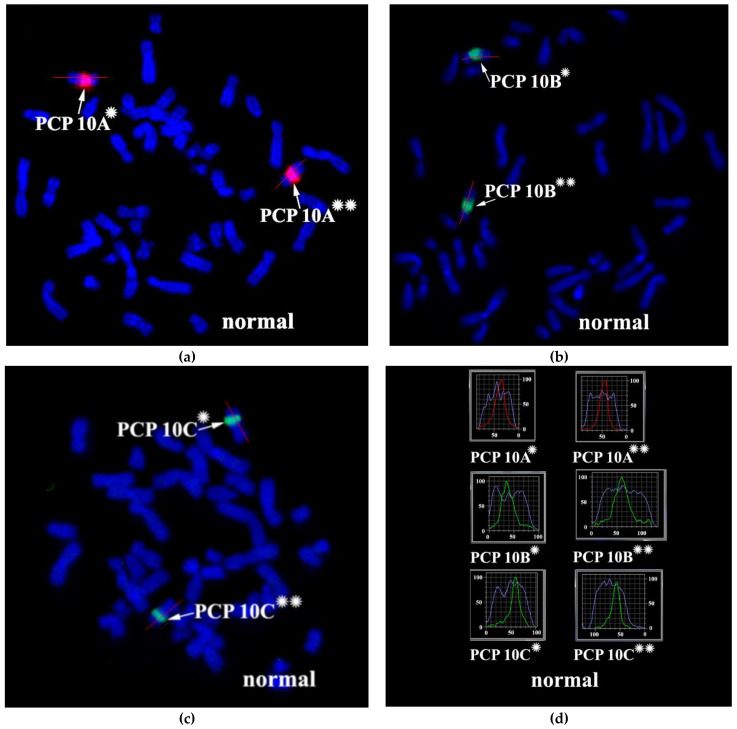
CISS hybridization of microdissected partial chromosome paints (PCPs; specific for three regions of human chromosome 10) with metaphase chromosomes of a healthy individual. (**a**) PCP10A (red signal), (**b**) PCP10B (green signal), and (**c**) PCP10C (green signal). (**d**) Fluorochrome profiles of homologous chromosome 10. The red line along the body of the chromosome is the line along which the profile of the relative signal intensity was determined. DAPI-stained chromosomes are blue. → indicates signals produced by the probe set on chromosome 10. * and ** mark the two homologous chromosomes 10.

Metaphase chromosome microdissection for PCP10A, PCP10B, and PCP10C was conducted and tested with CISS hybridization (Figure 2). We performed the microdissection of three overlapping regions of the q arm of the rearranged chromosome 10. The profile of the FISH signal across the region of dissection showed the highest intensity near the middle of the region of interest, and the signal intensity diminished toward its ends.

**Figure 3:**
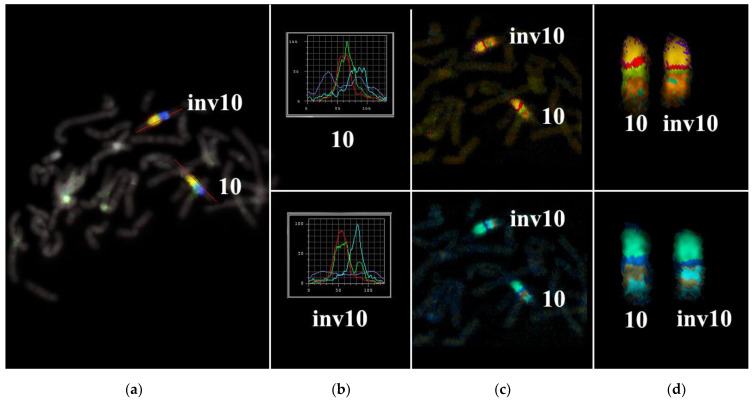
Multicolor FISH analysis of the long arm of chromosome 10 from the patient. (**a**) Three-color FISH of microdissected DNA probes [a combination of 10A (red), 10B (green), and 10C (blue)] labeled with different fluorochromes on metaphase chromosomes of the patient. The red line along chromosome 10 is the line along which the intensity profiles of hybridization signals were determined. (**b**) Profiles of signal intensities for abnormal [inv(10)] and normal chromosomes 10. (**c**,**d**) Different types of MCB pseudo-color classifiers are presented. inv10: chromosome 10 with the paracentric inversion in the q arm; 10: normal chromosome 10.

Metasystems Software developed for obtaining M-bands (MetaSystems Hard & Software GmbH, Altlussheim, Germany) converted the combination of mathematical ratios of signal intensity yielded by overlapping PCPs into pseudo-colors [20,21,22,26]. Therefore, pseudo-color bands were generated on the basis of the DNA content of corresponding chromosome regions. The results of CISS hybridization of three overlapping PCPs from the part of 10q were used for generating M-bands (Figure 3). Two classifiers of the pseudo-color M-bands were applied, and both revealed M-band patterns in the region of interest that were different between the long arm of chromosome 10 and in the long arm of chromosome inv(10) (Figure 3). They uncovered the inverted positioning of pseudo-color bands and changes in the size of some of them. Analyzing the M-band patterns of the rearranged chromosomes, we should take into account the metaphase chromosome spreading mechanism, which may affect M-band images. During metaphase chromosome spreading on a slide, DNA mixing of adjacent chromosome bands takes place. As a result, after CISS hybridization with microdissected DNA probes, a chromosome band emits signals generated by DNA from this band and from the adjacent bands. In rearranged chromosomes, in the region of the break and fusion, a new combination of adjacent bands arises that yields a new combination of signal intensity ratios in this region. This phenomenon is well known to investigators of chromosome translocations using 24-color FISH. In case of this FISH, the phenomenon could lead to new pseudo-color bands in the translocated chromosome on the border between regions derived from different chromosomes. With M-band generation, a similar problem may manifest itself in an analysis of inverted chromosomes [27]. In our study, the obtained M-band images showed an inversion in the long arm of the rearranged chromosome but did not allow to precisely locate the breakpoints that emerged during the inversion formation. This issue prompted us to analyze profiles of PCP signal intensity in detail.

**Figure 4:**
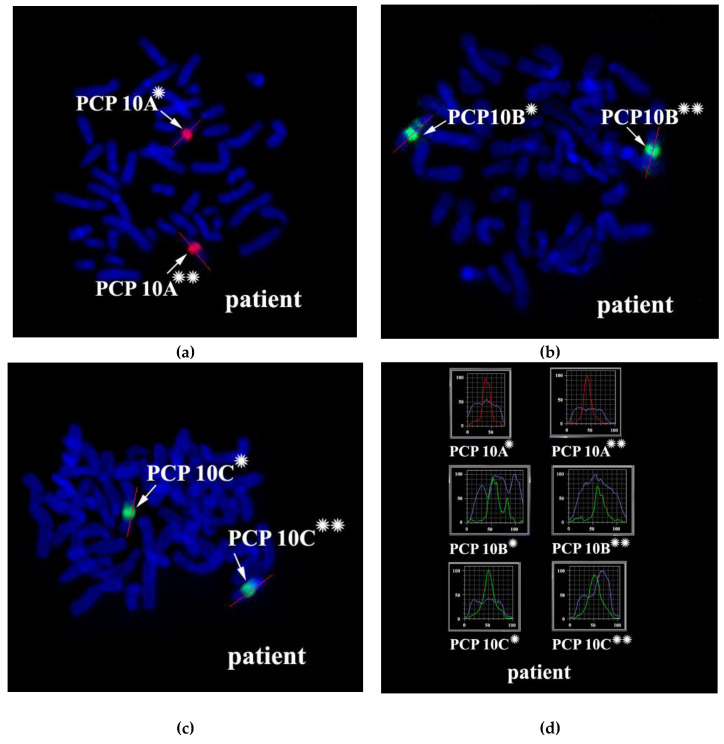
CISS hybridization of microdissected PCPs specific for two regions of human chromosome 10. (**a**) The PCP10A probe (TAMRA-dUTP, red signal); (**b**) the PCP10B probe (Alexa Fluor 488-dUTP, green signal); and (**c**) the PCP10C probe (DEAC-dUTP, green signal) with metaphase chromosomes of the patient. (**d**) Fluorochrome profiles of chromosome 10. An additional signal from PCP10B was found on the long arm of one of the patient’s chromosome 10 homologs. → indicates signals emitted by the PCP probes on chromosome 10. DAPI-stained chromosomes are blue. * and ** correspond to the normal and rearranged homologs, respectively.

The proximal borders of the regions painted with PCP10A (PCP10A^+^) and PCP10B (PCP10B^+^) appeared to be almost identical, but their distal borders were different. PCP10B painted near the PCP10A^+^ region a small additional distal region of 10q. A distal border of PCP10B^+^ proved to be located in the inverted region. In the inv(10) chromosome, the PCP10B-nonpainted region (PCP10B^−^) was found to be replaced with an inversion inside the PCP10B^+^ region disrupting it. It is worth noting that the replaced PCP10B^−^ region had a PCP10B signal intensity higher than the background level (Figure 3 and Figure 4). The replaced PCP10B^−^ region was small, and some DNA from adjacent PCP10B^+^ regions spread throughout it during metaphase chromosome preparation, thereby enhancing the intensity of the PCP10B FISH signal.

**Figure 5:**
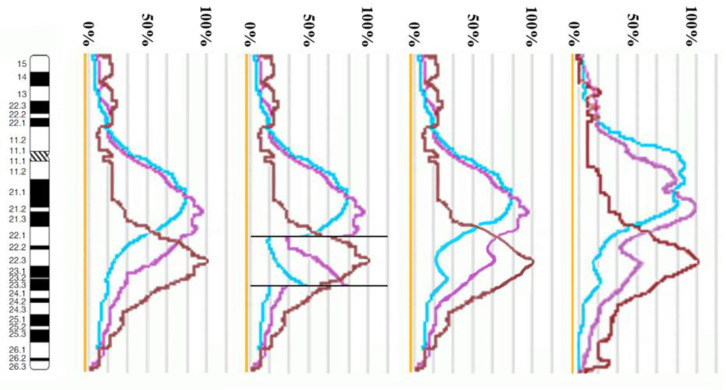
The scheme of hypothetical breakpoints of inverted regions in 10q22.1→q23.3 bands, as proposed on the basis of GTG-banding analysis.

**Figure 6:**
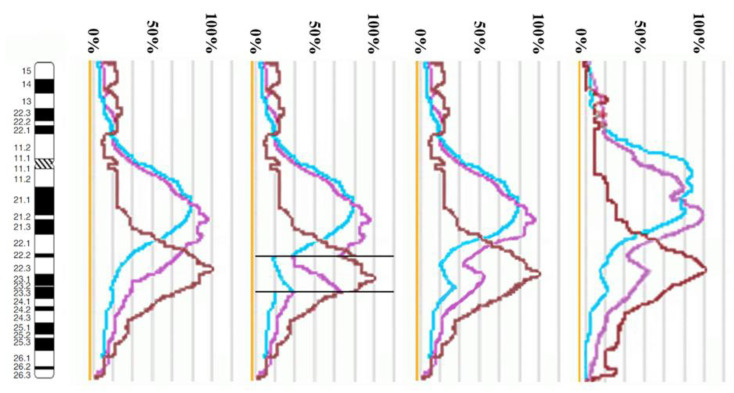
The scheme of hypothetical breakpoints of the inversion in 10q22.2→q23.3 bands, as proposed on the basis of GTG-banding analysis.

**Figure 7:**
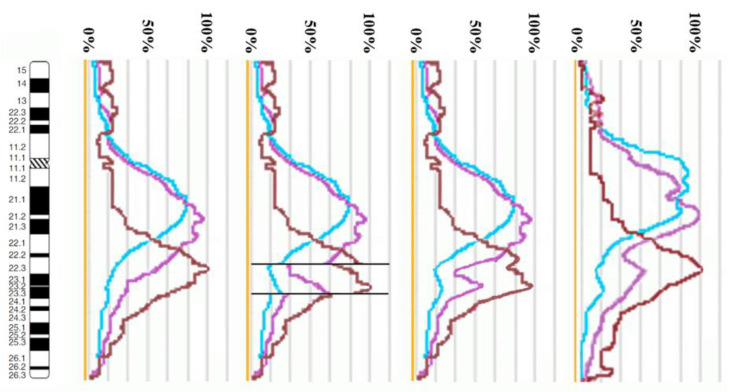
The scheme of hypothetical breakpoints of the inversion in 10q22.3→q23.3 bands, as proposed on the basis of GTG-banding analysis.

**Figure 8:**
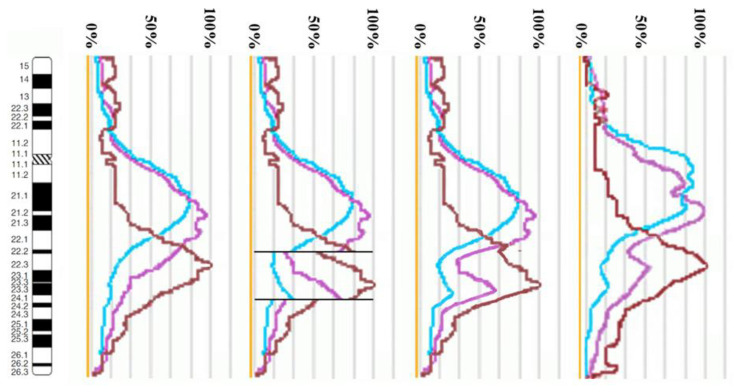
The scheme of hypothetical breakpoints of the inversion in 10q22.2→q24.1 bands, as proposed on the basis of GTG-banding analysis.

Using the profiles of signal intensity obtained with PCP10A, PCP10B, and PCP10C, we tested suppositions about different breakpoint locations by comparing reconstructed profiles with the one observed on chromosome inv(10). A reconstruction of signal intensity profiles was generated on the basis of signal profiles of the normal homolog. Profiles of signal intensity in the region of the presumed inversion were found to be inverted (Figure 5, Figure 6, Figure 7 and Figure 8). Taking into account DNA mixing between adjacent bands, we reconstructed expected profiles of FISH signal intensity for the regions of presumed breakpoints. In these regions, the higher intensity should go down due to DNA spreading to the adjacent band characterized by lower intensity, while lower intensity in this band should go up for the same reason. According to our experience, we adjusted the intensity profiles obtained after the presumed inversion in the analyzed regions (Figure 4, Figure 5, Figure 6 and Figure 7). At the proposed breakpoints, the signal intensity should show the average value of the new adjacent regions. We supposed that unchanged values of signal intensity should be observed at a distance equal to half of the band of medium size. A modified line of signal intensity passed from one such point to another one through the presumed point on the border of the inversion (Figure 5, Figure 6, Figure 7 and Figure 8). We tested the hypothetical breakpoints in bands presumed according to the results of GTG-banding analysis and in the adjacent bands. The findings of this analysis are presented in Figure 5, Figure 6, Figure 7 and Figure 8 for the four presumed inverted regions: 10q22.1→q23.3 (Figure 5), 10q22.2→q23.3 (Figure 6), 10q22.3→q23.3 (Figure 7), and 10q22.2→q24.1 (Figure 8). Reconstructed signal intensity profiles were compared with the one seen on chromosome inv(10). The best similarity was observed between profiles on chromosome inv(10) and reconstructed profiles of presumed inversion 10q22.2→q23.3. All peaks of signal intensity were located at the same position.

From the performed comparison, we deduced that the breakpoints are located in 10q22.2 and 10q23.3 subbands.

### 3.3. Looking for Small Microdeletions in the Breakpoint Regions of Inv(10)

**Figure 9:**
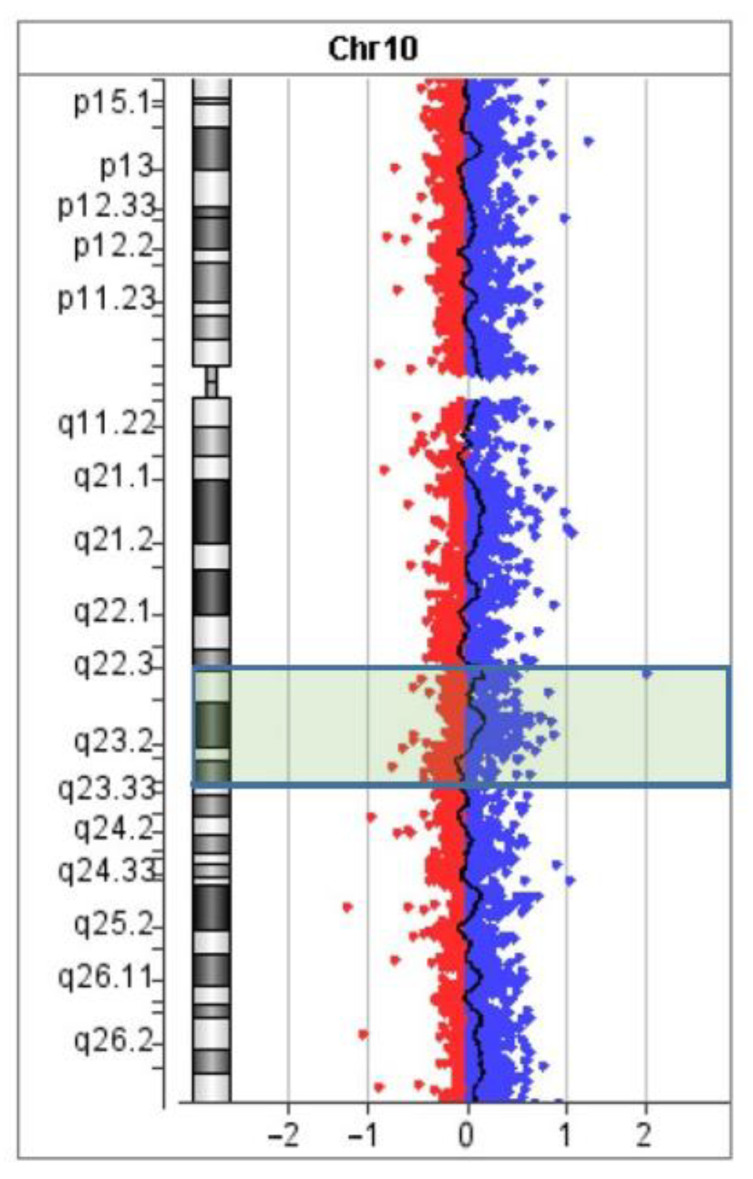
The aCGH profile of chromosome 10. The position of inverted region 10q22.2–q23.3 is highlighted.

DNA strand breaks and repair are common events in human cells. In any metaphase plate, several sister chromatid exchanges are usually seen [28]. This is a result of errors in DNA repair. Such errors may also lead to various chromosome rearrangements (e.g., inversions and translocations). Their frequency is associated with a longer time of existence of open DNA ends, and as a consequence, a greater chance of DNA damage. Small microdeletions may be some of the consequences of such a DNA lesion. Looking for microdeletions in the regions of inversion borders, we performed aCGH (Figure 9).

Although no deletion was detected, we should say that aCGH is based on the hybridization of unique genomic DNA fragments. It cannot detect possible alterations in repetitive DNA and chromosome regions enriched with repeats. The regions of the proposed breakpoints appeared to be enriched with repetitive DNA, which diminished the power of the aCGH analysis.

### 3.4. Microdissection and Single-Copy Chromosome Inv(10) Sequencing 

**Figure 10:**
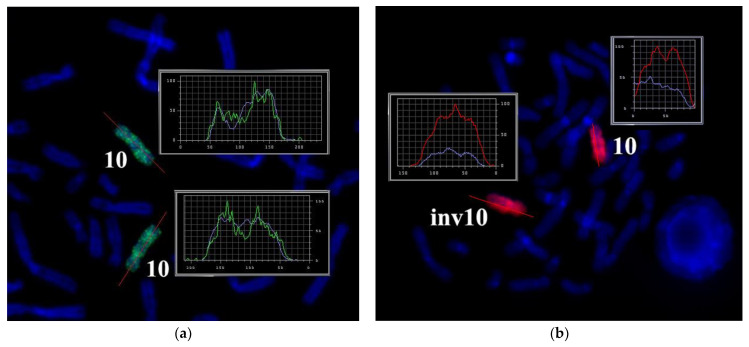
Suppression of FISH of the microdissected DNA probe of the abnormal chromosome (WCPinv10): (**a**) by metaphase chromosomes of a healthy individual (green signal) and (**b**) by metaphase chromosomes of the patient (red signal). *Labels.* 10: the normal homolog of chromosome 10; inv10: chromosome 10 containing the paracentric inversion in the long arm. Profiles of the intensity of the FISH signal and DAPI staining along chromosomes 10 and inv(10) are presented in the block. The intensity profiles of hybridization signals are determined relative to the line highlighted in red along chromosomes 10 and inv(10). Chromosomes were counterstained with DAPI (blue).

As an alternative approach to the search for microdeletions on chromosome inv(10), a microdissected DNA library was generated from this chromosome and then sequenced. To avoid the contamination of the microdissected DNA library with the DNA from normal chromosome 10, the DNA library was created from the only copy of chromosome inv(10). WCPinv(10) prepared on the basis of the resultant microdissected DNA library painted chromosome 10 and chromosome inv(10) completely. No specific signal was present on any other chromosome, and no unpainted region was noted on chromosomes 10 and inv(10). Nonetheless, a deletion of small regions on chromosome inv(10) could prevent detection by painting with a WCP owing to the DNA shuffling of adjacent regions (Figure 10).

**Figure 11:**
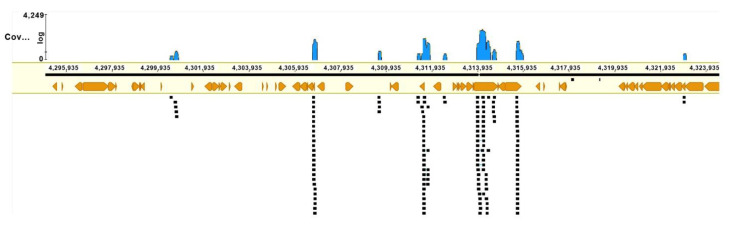
An example of mapping of reads in the region of the inversion. Black squares represent reads, and orange symbols denote repetitive DNA elements.

NGS was performed on the MiSeq platform (Illumina, San Diego, USA) with read length 2 × 150. Nevertheless, we should mention that the coverage with reads obtained by the sequencing of the microdissected DNA library appeared to be low (8.8%, 2,451,981 of 27,708,778 bp). Furthermore, the DNA library turned out to be enriched with repeats (Figure 11).

Accordingly, the obtained unique reads of the microdissected DNA library could serve only as markers of the chromosome regions and could not serve as an efficient tool for the detection of small deletions, especially in regions enriched with repeats. On the other hand, if the breakpoint was located in the sequences matching the DNA fragment present in the microdissected DNA library, then reads on its ends should be separated and should change their orientation. We looked for such cases in the region 10q22.1→q24.1, which includes an inversion and adjacent regions. The assembly of DNA sequences of this region contained 27 Mbp in ACC NC_000010.11, hg38. The resultant reads were mapped onto this region in the HISAT2 software and visualized with the GENEIOUS software.

**Figure 12:**
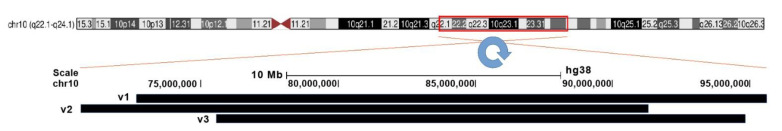
Three possible versions of inv(10) localization. The inverted region is marked by the red frame. Putative inverted regions are indicated by black bars: V1, V2, and V3.

After this procedure, we found a few dozen possible breakpoints in this region, but most of them corresponded to possible inversions less than 1 Mbp in size. Only three presumed inversions were similar in size to the inversion delineated by GTG-banding and the molecular cytogenetic technique (Table 1, Figure 12), and only one of them formed with breakpoints in the regions identified with multicolor FISH. 

Unfortunately, the presumed breakpoints proved to be located inside mobile elements and in the regions enriched with repeats. Such localization made the testing of the hypothetical breakpoints by PCR impossible. Nevertheless, these results allowed us to propose the most probable karyotype, 46,XY,inv(10)(q22.2q23.33), with the inversion in chromosome 10 located between positions 75,577,762 and 94,828,954 with a size of 19.251 Mbp.

## 4. Discussion

Polymorphic inversions are considered a type of structural variant that do not lead to a genomic imbalance with the location of the breakpoints within regions usually enriched with repetitive DNA. These features of inversion breakpoint regions make it difficult to examine them in detail. According to current knowledge, a possible effect of most of the paracentric inversions on the carrier phenotype is limited. Nonetheless, they may cause a disease by directly affecting gene structure or gene expression regulation in a different way. Paracentric inversions can also lead to a genomic imbalance in gametes in the case of an odd number of crossover events in the inverted region. On the other hand, in heterozygotes, recombination is inhibited by inversions [29], and such a crossover event can occur if the inverted region is long and constitutes more than 30% of the chromosome length [4]. The genomic imbalance deriving from crossing over inside an inverted region usually induces the early death of embryos, often before implantation.

The consequences of the inversion itself depend on the mechanism of its formation. Often, they are a result of nonallelic homologous recombination between inverted repeats. Nonetheless, double-strand break repair mechanisms involving nonhomologous end joining or replication-based mechanisms with fork stalling and template switching may also participate in inversion formation [30,31,32,33,34]. Usually, these mechanisms do not produce a considerable DNA gain or loss, but double-strand breaks followed by nonhomologous end joining can give rise to small deletions. Do such deletions have a substantial influence on the carrier phenotype? Can they be detected with routine diagnostic techniques?

In our project, we utilized modern molecular cytogenetic techniques and generated a microdissected DNA library followed by NGS for precisely describing an inversion in the long arm of chromosome 10. According to the findings, we proposed the most likely location of the breakpoints in the hg38 assembly as a reference genome. On the other hand, we cannot rule out that regions of the breakpoints in the original chromosome could differ from respective regions of 10q in hg38. Perhaps only NGS with long reads would allow us to describe the breakpoint regions of this patient’s chromosome in detail. Maybe in the future, improvements of long-read analysis will enable researchers to produce long reads of a single copy of an abnormal chromosome. Nevertheless, the following question will remain open: “Is the revealed difference in the breakpoint region from the reference genome a result of inversion formation or is it derived from normal genomic variation?” How can we use the data from the in-depth analysis of breakpoint regions for genetic counseling?

We suppose that, for efficient genetic counselling, the following problems should be resolved.

The first problem is associated with a possible gene disruption by the inversion. For the estimation of the possible significance of the chromosome rearrangement the integrity of genes in the regions of breakpoints should be tested. According to our analysis, no essential genes are located near the breakpoints, and consequently, one can expect that no gene is damaged. However, in other cases, the testing of gene integrity at the region of break-points should be performed.

The next possible important problem of genome disturbance could be associated with changes in gene transcription regulation in the breakpoint regions. However, the analysis of efficiency of gene expression regulation is more complicated. Regulatory elements can be replaced at a new position, or TAD structure may be altered. The estimation of the significance of such changes for phenotype formation in an inversion carrier seems to be a complex task. Furthermore, variation among human genomes—especially in the regions enriched with repeats—makes this task almost insurmountable. The best way to test the gene regulatory system is likely to direct analysis of the transcriptional activity of genes adjacent to the breakpoint regions with real-time or digital polymerase chain reaction. 

Attempts to find the answer by performing a comparison of the DNA sequences in the breakpoint regions and in corresponding region of reference genome can provide only suggestions of different levels of credibility. As pointed out previously, even small and submicroscopic inversions in such regions are difficult to distinguish from genomic variation, and all such inversions could be regarded as part of normal variation among humans [3,35]. Regarding this problem, Thomas Liehr et al. (2019) wrote earlier “For clinical impact and impact of large and small to submicroscopic paracentric inversions, the latter being part of normal variance in humans” [36]. Recently, we compared sequences from hg19 and hg38 in a region involved in small supernumerary marker chromosome formation and found numerous differences between them [37]. Comparative genomics of individual genomes has revealed a fantastic variation that includes—aside from single-nucleotide polymorphisms and copy number variants—a lot of structural chromosomal variation.

To answer the question “Are the sequences in the breakpoint regions a result of the inversion or an inversion followed by additional rearrangements?”, we should compare them with the relevant sequences in the unrearranged original chromosome. Nevertheless, it is quite possible that even such detailed analysis does not permit making a clinically significant conclusion about the impact of the analyzed inversion on the patient’s phenotype.

Besides the molecular cytogenomic techniques employed in this work and additionally discussed above, there is one that may also provide relevant information. HiC analysis can give additional information on breakpoint “location” in the reference genome assembly and about TAD chromosome structure. Unfortunately, the power of HiC analysis is low in regions enriched with repeats. Furthermore, a detected TAD disturbance can usually be converted into a diagnostic conclusion only in the form of a hypothesis.

The best approach in genome description in patients with balanced chromosome rearrangements is likely NGS with long-reads and de novo genome assembling. Unfortunately, even this approach cannot solve all problems. It also requires assembling a parent genome containing the chromosome of interest before its rearrangement. Its usual comparison with a reference genome does not allow for distinguishing some additional changes after chromosome rearrangement from new genomic variant.

Taking into account all the problems discussed above, we suppose that in diagnostics involving a paracentric inversion, it is necessary to pay attention to the following points: (a) the location of the breakpoints at the subband level at least; (b) the testing of the integrity and transcriptional activity of the genes located in the regions of the breakpoints; and (c) looking for deletions and duplications in the inverted regions and in the regions of the breakpoints. Knowledge about the precise location of breakpoints allows us to determine the region of interest for this subsequent analysis. The examination of gene integrity and transcriptional activity of these genes enables one to check for damage to the genes and their expression regulation. The aCGH can reveal a genomic imbalance associated with an inversion, but a small deletion of a region, especially a region enriched with repeats, can evade detection. We should mention that uneven crossover events inside an inverted region of a paracentric inversion depend on the size of the inverted region and typically lead to one dicentric chromosome and one acentric fragment. Such chromosome rearrangements usually yield such a substantial abnormality that some embryos are lost even before implantation [38].

**Figure 13:**
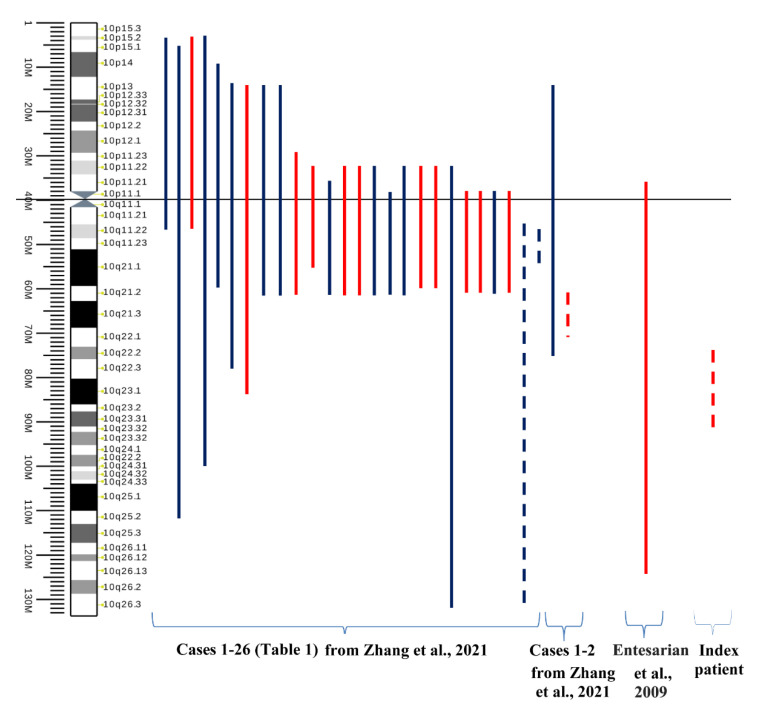
The map of chromosome 10 inversions in men. Red lines denote inversions detected in males with infertility, azoospermia, oligozoospermia, or recurrent miscarriage in the wife. Blue lines denote normal phenotype or cases with unavailble clinical data. Only four cases of paracentric inversion of 10q have been reported, including our case (dotted lines). (The source of the ideogram: https://commons.wikimedia.org/wiki/File:Human_chromosome_10_ideogram_vertical.svg accessed on 5 November 2022). “Cases 1–26” are reviewed cases in Table 1 from [39], “Cases 1–2” are own clinical observations from [39].

What can be recommended as key points for diagnostics of paracentric inversions? Small alterations in regions enriched with repeats may not be detected or distinguished from the normal variation of the human genome. We suppose that the most useful points for the diagnostics are perhaps the precise localization of breakpoints and the determination of the integrity and transcriptional activity of the genes located in regions of the breakpoints. Finally, we would like to state that paracentric inversions are rare events, and some of them have no pathogenic impact on their carriers (Figure 13) [39,40].

## 5. Conclusions

Hidden genomic abnormalities that take place in cases of ‘balanced chromosome rearrangements’ could lead to different problems including male infertility. Genetic counseling in such cases required complex comprehensive cytogenomic analysis. Nevertheless, even detail karyotype and genome description in carries of reciprocal translocations or inversions remain open questions mostly associated with the variability of personal genomes. The most efficient approach of genetic counseling in cases like those involved in the present study of paracentric inversion in the long arm of chromosome 10 should include the precise breakpoint mapping followed with testing integrity of genes located in the regions of breakpoints and analysis of their transcriptional activity. Genomic abnormality is only one of many possible reasons of male infertility. Consequently, the careful testing of individuals presenting with male infertility should include physical examination and thorough documentation of clinical history. It is essential to identify the cause of male infertility, and preimplantation genetic testing for structural rearrangements can be suggested to such couples.

## Figures and Tables

**Table 1:** Presumed breakpoints of inv(10) with genomic coordinates according to hg38.

No.	Start	End	Length	Bands	Repeat Family
1	72675611	95619369	22943758	10q22.1–10q23.33	L1P3
2	70631615	91322886	20691271	10q22.1–10q23.31	SVA_D
3	75577762	94828954	19251192	10q22.2–10q23.33	L1P3

## Data Availability

Raw data would be provided by corresponding authors upon request.
